# Dwell Time Allocation Algorithm for Multiple Target Tracking in LPI Radar Network Based on Cooperative Game

**DOI:** 10.3390/s20205944

**Published:** 2020-10-21

**Authors:** Chenyan Xue, Ling Wang, Daiyin Zhu

**Affiliations:** 1 Key Laboratory of Radar Imaging and Microwave Photonics, Ministry of Education, Nanjing University of Aeronautics and Astronautics, Nanjing 210016, China; xuechenyan@nuaa.edu.cn (C.X.); zhudy@nuaa.edu.cn (D.Z.); 2 Leihua Electronic Technology Research Institute, Aviation Industry Corporation of China, Wuxi 214063, China

**Keywords:** radar network, low probability of intercept (LPI), cooperative game, Nash bargaining solution (NBS), dwell time allocation

## Abstract

To solve the problem of dwell time management for multiple target tracking in Low Probability of Intercept (LPI) radar network, a Nash bargaining solution (NBS) dwell time allocation algorithm based on cooperative game theory is proposed. This algorithm can achieve the desired low interception performance by optimizing the allocation of the dwell time of each radar under the constraints of the given target detection performance, minimizing the total dwell time of radar network. By introducing two variables, dwell time and target allocation indicators, we decompose the dwell time and target allocation into two subproblems. Firstly, combining the Lagrange relaxation algorithm with the Newton iteration method, we derive the iterative formula for the dwell time of each radar. The dwell time allocation of the radars corresponding to each target is obtained. Secondly, we use the fixed Hungarian algorithm to determine the target allocation scheme based on the dwell time allocation results. Simulation results show that the proposed algorithm can effectively reduce the total dwell time of the radar network, and hence, improve the LPI performance.

## 1. Introduction

The dwell time management of radar is an important means to improve the Low Probability of Intercept (LPI) performance of a radar network. The existing research on time management of a radar network system focuses on the influence of the radiation interval, but ignores the beam dwell time. The transmitting parameters of digital array radar (DAR) networks can be dynamically controlled in the detection of targets. A reasonable selection of transmitting parameters including the dwell time, radiation interval, and transmitting power, which can improve the radar detection ability and the LPI performance. As to the time resource, both the radiation interval and the dwell time of a single radiation of DAR can be adjusted adaptively. Increasing the radiation interval of DAR and reducing the dwell time of a single radiation radar beam on the target are two main measures to improve the LPI performance of a DAR network.

The dwell time directly affects the signal to interference plus noise ratio (SINR) of the target, and hence, affect the tracking accuracy and the detection probability. In order to increase the SINR, the dwell time of the beam can be increased, but this simultaneously increases the probability of being detected by the passive detection system, which is unfavorable for maintaining the LPI performance of DAR networks.

In the target tracking by a DAR network, dwell time management refers to the joint management of the radar allocation mode and the duration of the dwell time. In ref. [1], the dwell time optimization was studied for the first time. Under the premise of meeting the given requirements on the target tracking performance, the radar time resource consumption was minimized. In ref. [2], a joint allocation method for beam pointing and dwell time was proposed for multiple target tracking. The expression of Bayesian Cramér-Rao Lower Bound (BCRLB) with regard to the dwell time was derived, from which the total dwell time can be minimized given the tracking accuracy of each target. In order to improve the radar system efficiency, a method of beam dwell time scheduling was proposed in ref. [3]. In ref. [4], the particle swarm optimization (PSO) algorithm was introduced to manage the dwell time of multiple sectors monitored by a multiple functional radar. An optimal monitoring area coverage along with an optimal detection probability can be achieved by an automatic allocation of the dwell time. In conclusion, the existing studies above put forward the idea of how to optimize the dwell time of radar beams and provide effective methods, but do not address well the dwell time optimization problem in the scenario of multiple target tracking by a radar network, especially the dwell time optimization by taking into account the LPI.

Game theory has been proven to be a powerful tool for the radar network resource allocation [5]. Game theory models can be divided into the non-cooperative game model and cooperative game model depending on the interaction between the decision makers in the radar network considered. In non-cooperative game models, each player acts in a selfish and rational way. For solving the problem of power allocation in radar networks, a wide range of non-cooperative game model algorithms have been proposed [6,7,8,9,10,11,12,13,14,15]. Zhou et al. [6,7,8,9] studied the problems of radio frequency (RF) congestion and shortage of RF spectrum resources. Through spectrum sharing between different wireless devices, it can alleviate the competition on the RF spectrum between radar and wireless communication systems. Gogineni et al. [10] designed a polarization waveform for the target detection by a distributed Multiple Input Multiple Output (MIMO) radar in the form of a two player zero sum game. No training data were needed. Song et al. [11] established a two player zero sum game model for the confrontation between targets and distributed MIMO radars. Piezzo et al. [12] proposed a radar network signal coding using the non-cooperative game model, in which each radar is regarded as participants in the game and the SINR performance of the radar network is maximized by optimizing the coding of the transmission signals of each radar. Bacci et al. [13] modeled a radar network with a rational player in a non-cooperative game, in which a power allocation algorithm was designed and a good trade-off between target detection performance and power could be achieved. Deligiannis et al. [14] studied the competitive power allocation problem of MIMO Radar considering the multiple target jamming, which was modeled as a non-cooperative game to minimize the total transmit power of the radar for a given detection threshold. Deligiannis et al. [15] studied the power allocation strategy in the framework of non-cooperative game theory. The Nash equilibrium (NE) was carried out for the Multistatic MIMO radar network.

Compared with cooperative game theory, the non-cooperative game theory method degrades the system performance [5], because each participant in the non-cooperative game acts in a selfish and rational way to maximize their own success. Thus, the cooperative game model is expected to be more suitable for resource allocation in radar networks. The Nash bargaining solution (NBS) is a promising candidate solution. Most existing research focuses on finding the NBS for the transmission power allocation. Sun et al. [16] used game theory to model the optimal resource management in the problem of target localization. An optimal power allocation algorithm was proposed based on the cooperative game theory. The Shapley value was used to represent the contribution of each radar transmitter. Monte Carlo simulation results showed that the algorithm can provide higher target localization accuracy than the uniform power allocation method. Chen et al. [17] proposed a distributed power management method for synchronous and asynchronous network cooperative location and derive the NBS of power allocation for cooperative positioning. In addition, Chen et al. [18] extended the formula in [16] to the target tracking problem by introducing a motion model of the target.

With the rapid development of advanced interceptors, the LPI design has become an important part of modern radar systems [19,20,21]. The radar dwell time resources must be reduced as much as possible for good LPI performance. Shi et al. [22] proposed a power allocation method considering the LPI of the radar network using the idea of cooperative game. The LPI performance is improved by minimizing the total transmit power constrained by predetermined target detection. A new network utility function based on SINR is defined and used as an index to evaluate the power allocation performance. The existence and uniqueness of NBS in the proposed cooperative power allocation model is proved. The influence of the relative geometry of the target and radar and the radar cross section (RCS) of the target on the power allocation are analyzed in detail.

In conclusion, the resource management of radar networks based on game theory has gained much interest, but the dwell time optimization of the radar network for multiple target tracking is not considered, especially for LPI radar networks. Thus, how to optimize the allocation of radar dwell time with better LPI performance needs to be addressed as a key part of the resource management of LPI radar networks.

In this paper, we focus on the dwell time management of an LPI radar network operating for multiple target tracking. We propose a dwell time allocation algorithm based on the cooperative game theory. Two variables, including the dwell time and target allocation indicators, are optimized. The dwell time allocation and the target allocation are treated as two subproblems. Firstly, a comprehensive utility function accounting for the target detection performance is designed. A model for the dwell time allocation of the radar network exploiting NBS is established. The Lagrange relaxation algorithm is used to obtain the optimal dwell time strategy of each radar. The Newton iteration method is used to obtain the iterative formula for the dwell time of each radar. Consequently, the optimal dwell time allocation for each target is obtained. Secondly, according to the dwell time allocation results, the fixed Hungary algorithm is used to determine the target allocation schemes.

The organization of the rest of the paper is as follows: In Section 2, the target motion model for a multi-target scenario is presented and the received signals of the radar network are formulated. In Section 3, the concept of cooperative game theory is briefly elaborated followed by the design of a comprehensive utility function in terms of target detection performance. Then, a radar network dwell time allocation model exploiting NBS is established. Combining the Lagrange relaxation algorithm with the Newton iteration method, the iterative formula for the dwell time of each radar is derived. Lastly, based on the assigned dwell time, the optimization problem of radar selection is studied to minimize the total dwell time of radar network. Section 4 demonstrates the dwell time allocation algorithm by extensive simulation experiments. Section 5 draws a conclusion.

## 2. Signal Model for Radar Networks

### 2.1. Target Motion Model

Suppose there are Q sparse distributed targets in the three-dimensional plane, and all the targets move along a straight trajectory. The model of the $q^{\mathrm{th}}$ moving target is expressed as follows:

(1) $$X_{k}^{q}=FX_{k-1}^{q}+W^{q}$$

where $X_{k}^{q}={\left[x_{k}^{q},{\dot{x}}_{k}^{q},y_{k}^{q},{\dot{y}}_{k}^{q},z_{k}^{q},{\dot{z}}_{k}^{q}\right]}^{T}$ denotes the state vector of the $q^{\mathrm{th}}$ target at the $k^{\mathrm{th}}$ time instant, where $\left(x_{k}^{q},y_{k}^{q},z_{k}^{q}\right)$ and $\left({\dot{x}}_{k}^{q},{\dot{y}}_{k}^{q},{\dot{z}}_{k}^{q}\right)$ are the position and velocity of the $q^{\mathrm{th}}$ target at the $k^{\mathrm{th}}$ time instant, respectively. $W^{q}$ is the process noise of the $q^{\mathrm{th}}$ target. F is the target state transition matrix:

(2) $$F=I_{3}⊗\left[\begin{array}{cc} 1 & T\\ 0 & 1 \end{array}\right]$$

where T is the sampling interval. ⊗ is the operation symbol of Kronecker product. $I_{3}$ is the identity matrix of 3×3.

### 2.2. Received Signal Model

Suppose that there are N radars in the radar network and each radar performs the detection independently. For the $i^{\mathrm{th}}$ radar, the received signal is expressed as follows [23]:

(3) $$s_{i,q,k}=\chi_{i,q,k}\sqrt{P_{i,q,k}}x_{i,q,k}+\sum_{j=1,j\neq i}^{N}\zeta_{i,j,q,k}\sqrt{P_{j,q,k}}x_{j,q,k}+w_{i,q,k}$$

where the first term represents the component due to the $i^{\mathrm{th}}$ radar own transmission, the second terms represents component due to the other radars’ transmission and the last term represents the noise. Note that subscripts i, q, k denotes the indices of the radar, target, and time instant, respectively. More specifically, $s_{i,q,k}$ denotes the sample of the received signal at the $k^{\mathrm{th}}$ time instant from the $q^{\mathrm{th}}$ target due to the $i^{\mathrm{th}}$ radar transmission, $x_{i,q,k}$ denotes the transmitted waveform of the $i^{\mathrm{th}}$ radar irradiating the $q^{\mathrm{th}}$ target at the $k^{\mathrm{th}}$ time instant, $P_{i,q,k}$ and $P_{j,q,k}$ are the transmitting power of the $i^{\mathrm{th}}$ radar and $j^{\mathrm{th}}$ radar, respectively, $\chi_{i,q,k}\sim \mathrm{CN}\left(0,h_{i,q,k}^{t}\right)$ denotes the path gain between the $i^{\mathrm{th}}$ radar and the $q^{\mathrm{th}}$ target at the $k^{\mathrm{th}}$ time instant, $\zeta_{i,j,k}\sim \mathrm{CN}\left(0,c_{i,j,k}h_{i,j,q,k}^{d}\right)$ denotes the cross path gain between the $i^{\mathrm{th}}$ radar and the $j^{\mathrm{th}}$ radar, and $w_{i,q,k}\sim \mathrm{CN}\left(0,\sigma^{2}\right)$ denotes the Gaussian white noise with zero mean value and $\sigma^{2}$ variance at the $i^{\mathrm{th}}$ radar receiver. Note that $h_{i,q,k}^{t}$ denotes the variance of path gain of the $i^{\mathrm{th}}$ radar and the $q^{\mathrm{th}}$ target, $c_{i,j,k}h_{i,j,k}^{d}$ denotes the variance of path gain of the $i^{\mathrm{th}}$ radar and the $j^{\mathrm{th}}$ radar, and $c_{i,j,k}$ denotes the cross correlation coefficient between the $i^{\mathrm{th}}$ radar and the $j^{\mathrm{th}}$ radar at the $k^{\mathrm{th}}$ time instant. The variance of the corresponding path gain is defined as follows [23]:

(4) $$\left.\begin{array}{c} h_{i,q,k}^{t}=\frac{G_{t}G_{r}\sigma_{i,q}^{\mathrm{RCS}}\lambda^{2}}{{\left(4\pi \right)}^{3}R_{i,q,k}^{4}}\\ h_{i,j,q,k}^{d}=\frac{G_{t}^{\prime }G_{r}^{\prime }\lambda^{2}}{{\left(4\pi \right)}^{2}d_{i,j}^{2}} \end{array}\right\}$$

where $G_{t}$ denotes the main lobe gain of the radar transmitting antenna, $G_{r}$ denotes the main lobe gain of the radar receiving antenna, $G_{t}^{\prime }$ denotes the side lobe gain of the radar transmitting antenna, $G_{r}^{\prime }$ denotes the side lobe gain of the radar receiving antenna, $\sigma_{i,q}^{\mathrm{RCS}}$ denotes the radar cross section (RCS) of the $q^{\mathrm{th}}$ target relative to the $i^{\mathrm{th}}$ radar, λ denotes the wavelength of radar transmitting signal, $R_{i,q,k}$ denotes the distance between the $i^{\mathrm{th}}$ radar and the $q^{\mathrm{th}}$ target at the $k^{\mathrm{th}}$ time instant, and $d_{i,j}$ denotes the linear distance between the $i^{\mathrm{th}}$ radar and the $j^{\mathrm{th}}$ radar. All path gains are assumed to be constant over the dwell time of the radar beam.

The missed detection probability $P_{i,q,k}^{\mathrm{MD}}\left(\delta_{i,q,k},\gamma_{i,q,k}\right)$ and false alarm probability $P_{i,q,k}^{\mathrm{FA}}\left(\delta_{i,q,k}\right)$ of the $i^{\mathrm{th}}$ radar are defined as [23]:

(5) $$\left.\begin{array}{l} p_{i,q,k}^{\mathrm{MD}}\left(\delta_{i,q,k},\gamma_{i,q,k}\right)=1-{\left(1+\frac{\delta_{i,q,k}}{1-\delta_{i,q,k}}\cdot \frac{1}{1+\gamma_{i,q,k}n}\right)}^{1-n}\\ p_{i,q,k}^{\mathrm{FA}}\left(\delta_{i,q,k}\right)={\left(1-\delta_{i,q,k}\right)}^{n-1} \end{array}\right\}$$

where $\delta_{i,q,k}$ denotes the system detection threshold and n denotes the number of pulses received within the radar dwell time.

The energy of the $i^{\mathrm{th}}$ radar received signal is affected by the dwell time of the $i^{\mathrm{th}}$ radar transmission, and the jamming energy of the $i^{\mathrm{th}}$ radar received signal is affected by the dwell time of the other radar detection targets. The numerator of the SINR describes the return signal scattered off the target, while the denominator consists of the interference and noise. The return signal scattered off the target is $h_{i,q,k}^{t}t_{i}{}_{,q,k}^{}$. The total interference plus noise received by the $i^{\mathrm{th}}$ radar is $\sum_{j=1,j\neq i}^{N}c_{i,j}h_{i,j,k}^{d}t_{j}{}_{,q,k}^{}+\sigma^{2}$. Thus, $\gamma_{i,q,k}$ denotes the SINR value of the $i^{\mathrm{th}}$ radar received echo, which is defined as:

(6) $$\gamma_{i,q,k}=\frac{h_{i,q,k}^{t}t_{i}{}_{,q,k}^{}}{\sum_{j=1,j\neq i}^{N}c_{i,j}h_{i,j,k}^{d}t_{j}{}_{,q,k}^{}+\sigma^{2}}$$

where $t_{i,q,k}^{}$ denotes the dwell time of the $q^{\mathrm{th}}$ target irradiated by the $i^{\mathrm{th}}$ radar at the $k^{\mathrm{th}}$ time instant. Equation (6) can also be written as:

(7) $$\gamma_{i,q,k}=\frac{h_{i,q,k}^{t}t_{i,q,k}^{}}{I_{-i,q,k}}$$

where $I_{-i,q,k}$ denotes the total interference plus noise received by the $i^{\mathrm{th}}$ radar:

(8) $$I_{-i,q,k}=\sum_{j=1,j\neq i}^{N}c_{i,j}h_{i,j,k}^{d}t_{j,q,k}^{}+\sigma^{2}$$

In order to ensure its target detection performance, the received SINR of the $i^{\mathrm{th}}$ radar should not be less than the pre-defined minimum value $\gamma_{\mathrm{th}}^{\min}$. Given $p_{i,q,k}^{\mathrm{MD}}\left(\delta_{i,q,k},\gamma_{i,q,k}\right)$ and $p_{i,q,k}^{\mathrm{FA}}\left(\delta_{i,q,k}\right)$, the SINR value received by each radar can be obtained.

### 2.3. Schleher Intercept Factor

The equation of radar system can be written as follows:

(9) $$R_{rad}^{4}=K_{rad}\frac{t_{i,q,k}}{SNR_{rad}}$$

where:

(10) $$K_{rad}=\frac{P_{t}G_{t}G_{r}\sigma_{t}\lambda^{2}}{{\left(4\pi \right)}^{3}KT_{o}B_{r}F_{r}L}$$

where $P_{t}$ is the transmitter power of the radar, $\sigma_{t}$ is the RCS of target, K is the Boltzmann constant, $T_{0}$ is the radar receiver’s noise temperature, $B_{r}$ is the bandwidth of radar receiver matched filter, $F_{r}$ is the noise coefficient of radar receiver, and $R_{i,q,k}^{}$ is the distance from the $i^{\mathrm{th}}$ radar to the $q^{\mathrm{th}}$ target. L is the loss coefficient of radar system and $SNR_{rad}$ is the SNR of the radar. For the interception receiver, the equation is as follows:

(11) $$R_{int}^{2}=K_{int}\frac{t_{i,q,k}}{SNR_{int}}$$

where:

(12) $$K_{int}=\frac{P_{t}G_{t}G_{i}\lambda_{}^{2}}{{\left(4\pi \right)}^{2}KT_{o}B_{i}F_{i}L_{i}}$$

where $SNR_{int}$ is the SNR of the signal processor input of the interception receiver, $G_{i}$ is the antenna gain of the interception receiver, $B_{i}$ is the bandwidth of the interception receiver, $F_{i}$ is the noise Fi of the interception receiver, $L_{i}$ is the system loss coefficient from the radar antenna to the interception receiver, and $R_{int}$ is the distance from the radar network system to the interception receiver. Here, the Schleher interception factor is used to characterize the LPI performance of the radar network system. The Schleher interception factor can be calculated by the following formula:

(13) $$\alpha =\frac{R_{int}}{R_{rad}}$$

where $R_{rad}$ is the maximum detection range of the radar and $R_{int}$ is the maximum intercept distance of the interception receiver. From Equation (13), we can calculate the Schleher interception factor of radar network system as follows:

(14) $$\alpha_{i,q,k}=\frac{R_{int}}{R_{rad}}={\left(\frac{K_{int}^{2}\cdot SNR_{rad}}{K_{rad}\cdot SNR_{int}^{2}}\right)}^{1/4}\cdot {\left(t_{i,q,k}\right)}^{1/4}$$

where:

(15) $$C_{1}={\left(\frac{K_{int}^{2}\cdot SNR_{rad}}{K_{rad}\cdot SNR_{int}^{2}}\right)}^{1/4}$$

According to the definition of Schleher interception factor, when $\alpha_{i,q,k}>1$, the intercept receiver can detect the signal transmitted by radar; when $\alpha_{i,q,k}\leq 1$, the radar can detect the target, but the intercept receiver cannot detect the signal transmitted by the radar. Therefore, when $\alpha_{i,q,k}\leq 1$, the radar is in RF stealth state. In addition, with the decrease of dwell time $t_{i,q,k}$, the smaller the Schleher intercept factor is, the better the LPI performance of radar system is.

The Schleher interception factor substituted into Equation (6) and Equation (7). It can also be written as:

(16) $$\gamma_{i,q,k}=\frac{h_{i,q,k}^{t}{\left(\alpha_{i,q,k}\right)}^{4}}{I_{-i,q,k}{\left(C_{1}\right)}^{4}}$$

where:

(17) $$I_{-i,q,k}=\sum_{j=1,j\neq i}^{N}c_{i,j}h_{i,j,k}^{d}{\left(\frac{\alpha_{j,q,k}}{C_{1}}\right)}^{4}+\sigma^{2}$$

## 3. Dwell Time Allocation Algorithm for Radar Networks Based on a Cooperative Game Model

The genetic algorithm (GA) uses selection, crossover, and mutation operators to search the most optimal solution. The global searchability is strong, but the local searchability is weak. Generally, only the sub-optimal solution of the problem can be obtained, not the optimal solution. Classical nonlinear programming algorithms mostly adopt gradient descent to solve the problem, with strong local searchability but weak global searchability [24]. This paper adopts cooperative game models to avoid the shortcomings of GA’s weak local searchability and the nonlinear programming algorithm’s weak global searchability.

In the non-cooperative game theory, each player chooses appropriate strategy behaviors to maximize their own utility [25]. In the process of selecting strategy behavior, each participant considers the influence of each other’s strategy choice, which belongs to selfish and irrational behavior. Although it can ultimately make each participant reach the Nash equilibrium state, the overall efficiency of the system is not optimal.

Compared with non-cooperative game theory, cooperative game theory refers to the way in which game participants adopt alliance and cooperation in the game [26]. In a cooperative game model, each participant adopts the method of compromise and bargaining, so that each player reaches a cooperative agreement. Compared with the non-cooperative game, the cooperative game pays more attention to the overall optimal strategy, which can increase the overall performance of the system.

In this paper, the cooperative game theory and NBS are applied to the dwell time management for in the target tracking by a LPI radar network. The optimal strategy of dwell time allocation of radar network is studied.

### 3.1. Optimization of Dwell Time Allocation Using Nash Bargaining Solution

The cooperative game is mainly composed of two parts: all game participants, and the overall utility function. The set of participants in the cooperative game can be expressed as $\Phi =\left\{1,2,\cdots ,N\right\}$, where N denotes the number of radars in the radar network. Let $s_{i,q,k}^{*}$ denote the NBS of the maximum utility function $u_{i,q,k}(t_{i,q,k},T_{-i,q,k})$ in cooperative games. It is given by [23]:

(18) $$s_{i}^{*}=\arg\;\max\prod_{i=1}^{N}u_{i,q,k}\left(t_{i,q,k},T_{-i,q,k}\right)$$

where $T_{-i,q,k}=\left\{t_{j,q,k}^{\min}\in S,\forall j\in \Phi ,j\neq i\right\}$ denotes the dwell time set of radars except the $i^{\mathrm{th}}$ radar. S denotes the payment set obtained by game participants after cooperation. Since the SINR $\gamma_{i,q,k}$ can well represent the target detection performance of each radar in the radar network system, the utility function $u_{i,q,k}(t_{i,q,k},T_{-i,q,k})$ of cooperative game can be expressed by a certain function form of SINR. The utility function is the physical quantity of SINR measured by the $i^{\mathrm{th}}$ radar when it detects targets with dwell time $t_{i,q,k}$. The utility function $u_{i,q,k}(t_{i,q,k},T_{-i,q,k})$ of cooperative game involves SINR $\gamma_{i,q,k}$ of received echo. $\gamma_{i,q,k}$ represents SINR value of the $i^{\mathrm{th}}$ radar received echo. $\gamma_{i,q,k}$ is a function of Schleher interception factor $\alpha_{i,q,k}$. The Schleher interception factor $\alpha_{i,q,k}$ is a function of dwell time $t_{i,q,k}$. According to the Nash theorem, the comprehensive utility function in a cooperative game model is defined as follows:

(19) $$V_{i,q,k}\left(t_{i,q,k},T_{-i,q,k}\right)=\prod_{i=1}^{N}u_{i,q,k}\left(t_{i,q,k},T_{-i,q,k}\right)=\prod_{i=1}^{N}\frac{\gamma_{i,q,k}-\gamma_{\mathrm{th}}^{\min}}{\gamma_{i,q,k}}$$

It can be seen from Equation (19) that the cooperative game model contains N players, and the strategic behavior set of the $i^{\mathrm{th}}$ player is $S_{i,q,k}=\{t_{i,q,k}|t_{i,q,k}\in [T_{}^{\min},T_{}^{\max}]\}$. Under the condition that the total dwell time is less than the maximum dwell time $T_{}^{\max}$ of the system, each radar achieves the maximum utility function by bargaining with each other.

The main purpose of this paper is to minimize the total dwell time of radar network system under certain target detectability constraints. In this paper, SINR is used to characterize the target detection performance of the system, and a new comprehensive utility function based on SINR is proposed by introducing the path gain parameter $h_{i,q,k}^{t}$. Equation (19) can be transformed into the following form:

(20) $$V_{i,q,k}\left(t_{i,q,k},T_{-i,q,k}\right)=\prod_{i=1}^{N}{\left(\frac{\gamma_{i,q,k}-\gamma_{\mathrm{th}}^{\min}}{\gamma_{i,q,k}}\right)}^{h_{i,q,k}^{t}}$$

Therefore, considering that the logarithmic utility function can guarantee that the feasible utility space is a convex set [23]. A dwell time allocation model based on NBS is constructed as follows:

(21) $$\left.\begin{array}{ccc} & \underset{t_{i,q,k},i\in \Phi }{\max}\sum_{q=1}^{Q}\sum_{i=1}^{N}h_{i,q,k}^{t}\ln\left(\frac{\gamma_{i,q,k}-\gamma_{\mathrm{th}}^{\min}}{\gamma_{i,q,k}}\right), & \\ s.t. & \gamma_{i,q,k}\geq \gamma_{\mathrm{th}}^{\min},i\in \Phi & \\ & \left\{\begin{array}{ccc} t_{i,q,k}=0 & \mathrm{if} & v_{i,q,k}^{}=0\\ T_{}^{\min}\leq t_{i,q,k}\leq T_{}^{\max},i\in \Phi & \mathrm{if} & v_{i,q,k}^{}=1 \end{array}\right. & \\ & \sum_{i=1}^{Q}v_{i,q,k}^{}\leq 1,\sum_{i=1}^{N}v_{i,q,k}^{}=1 & \end{array}\right\}$$

where $v_{i,q,k}^{}=1$ denotes that the $i^{\mathrm{th}}$ radar irradiates the $q^{\mathrm{th}}$ target, $v_{i,q,k}^{}=0$ means that the $i^{\mathrm{th}}$ radar does not irradiate the $q^{\mathrm{th}}$ target at the $k^{\mathrm{th}}$ time instant, $\gamma_{\mathrm{th}}^{\min}$ denotes the SINR threshold of target detection performance, $T_{}^{\max}$ denotes the maximum dwell time of the $i^{\mathrm{th}}$ radar. $\sum_{q=1}^{Q}v_{i,q,k}^{}\leq 1$ means that each radar can track at most one target, and $\sum_{i=1}^{N}v_{i,q,k}^{}=1$ means that each target is tracked by one radar at the $k^{\mathrm{th}}$ time instant.

It should be noted that parameter $h_{i,q,k}^{t}$ is a function related to the distance between the $i^{\mathrm{th}}$ radar and the $q^{\mathrm{th}}$ target. By introducing the parameter $h_{i,q,k}^{t}$, the dwell time can be reduced when the SNR required for reliable target detection is reduced. However, the target detection SINR requirement of each radar can still be guaranteed. It can be seen from the simulation results in Section 4 that the dwell time allocation result of a radar network system is mainly determined by the relative position of the target to each radar. In the process of target detection, the dwell time resource tends to be allocated to the radar, which is far away from the target, so as to minimize the total dwell time of the system to improve its LPI performance on the premise of ensuring the detection performance of each radar target.

### 3.2. Iterative Algorithm for Dwell Time Allocation

The dwell time allocation is a nonconvex optimization problem with two variables, $t_{i,q,k}$ and $v_{i,q,k}$, as shown in Equation (21). The common algorithm to solve this problem is to optimize the two variables by a two-step decomposition method [27]. Firstly, the optimal dwell time strategy of each radar is determined by the Lagrange relaxation algorithm. The dwell time iteration formula of each radar is obtained by using the Newton iteration method. Consequently, the dwell time allocation of radar corresponding to each target is obtained. Secondly, according to the dwell time allocation results, the fixed Hungary algorithm is used to determine the target allocation schemes.

For the $q^{\mathrm{th}}$ target, the optimization problem of Equation (21) can be rewritten to the form containing only variable $t_{i,q,k}^{}$ for a given constraint condition $\sum_{i=1}^{N}v_{i,q,k}^{}=1$:

(22) $$\begin{array}{cc} & \underset{t_{i,q,k},i\in \Phi }{\max}\sum_{i=1}^{N}h_{i,q,k}^{t}\ln\left(\frac{\gamma_{i,q,k}-\gamma_{\mathrm{th}}^{\min}}{\gamma_{i,q,k}}\right)\\ s.t. & \left\{\begin{array}{c} \gamma_{i,q,k}\geq \gamma_{\mathrm{th}}^{\min},i\in \Phi \\ T_{}^{\min}\leq t_{i,q,k}\leq T_{}^{\max},i\in \Phi \end{array}\right. \end{array}$$

Equation (22) is an optimization problem with multiple constraints, which is solved by the Lagrange relaxation algorithm. By introducing Lagrange multipliers ${\left\{{\left(\eta_{i,q,k}\right)}^{\left(l\right)}\right\}}_{i=1}^{N}$, ${\left\{{\left(\mu_{i,q,k}\right)}^{\left(l\right)}\right\}}_{i=1}^{N}$, and ${\left\{{\left(\psi_{i,q,k}\right)}^{\left(l\right)}\right\}}_{i=1}^{N}$, Equation (22) can be transformed into:

(23) $$\begin{array}{l} L\left({\left\{u_{i,q,k}\left(t_{i,q,k},T_{-i,q,k}\right)\right\}}_{i=1}^{N},{\left\{\eta_{i,q,k}\right\}}_{i=1}^{N},{\left\{\mu_{i,q,k}\right\}}_{i=1}^{N},{\left\{\psi_{i,q,k}\right\}}_{i=1}^{N}\right)\\ =\sum_{i=1}^{N}h_{i,q,k}^{t}\ln\left(\frac{\gamma_{i,q,k}-\gamma_{\mathrm{th}}^{\min}}{\gamma_{i,q,k}}\right)-\eta_{i,q,k}\left(\gamma_{i,q,k}-\gamma_{\mathrm{th}}^{\min}\right)+\mu_{i,q,k}\left(t_{i,q,k}-T^{\max}\right)-\psi_{i,q,k}\left(t_{i,q,k}-T^{\min}\right) \end{array}$$

Find the first-order partial derivative of Equation (23) with respect to $t_{i,q,k}$, and let $\partial L/\partial t_{i,q,k}=0$, then:

(24) $$\begin{array}{ll} \frac{\partial L}{\partial t_{i,q,k}} & =\frac{h_{i,q,k}^{\text{t}}}{\gamma_{i,q,k}-\gamma_{\text{th}}^{\min}}\cdot \frac{\frac{\partial \gamma_{i,q,k}}{\partial t_{i,q,k}}\gamma_{\text{th}}^{\min}}{\gamma_{i,q,k}^{2}}-\eta_{i,q,k}\frac{\partial \gamma_{i,q,k}}{\partial t_{i,q,k}}+\mu_{i,q,k}-\psi_{i,q,k}\\ & =\frac{h_{i,q,k}^{\text{t}}\gamma_{i,q,k}}{\gamma_{i,q,k}-\gamma_{\text{th}}^{\min}}\cdot \frac{\frac{\gamma_{i,q,k}}{t_{i,q,k}}\gamma_{\text{th}}^{\min}}{\gamma_{i,q,k}^{2}}-\eta_{i,q,k}\frac{\partial \gamma_{i,q,k}}{\partial t_{i,q,k}}+\mu_{i,q,k}-\psi_{i,q,k}\\ & =\frac{h_{i,q,k}^{\text{t}}\gamma_{\text{th}}^{\min}}{t_{i,q,k}\left(\gamma_{i,q,k}-\gamma_{\text{th}}^{\min}\right)}-\eta_{i,q,k}\frac{\gamma_{i,q,k}}{t_{i,q,k}}+\mu_{i,q,k}-\psi_{i,q,k}\\ & =0 \end{array}$$

Substituting Equation (7) into Equation (24), we can get the following results:

(25) $$\begin{array}{l} t_{i,q,k}\left(\frac{h_{i,q,k}^{t}t_{i,q,k}}{I_{-i,q,k}}-\gamma_{\mathrm{th}}^{\min}\right)=\frac{h_{i,q,k}^{t}\gamma_{\mathrm{th}}^{\min}}{\eta_{i,q,k}\frac{\gamma_{i,q,k}}{t_{i,q,k}}-\mu_{i,q,k}+\psi_{i,q,k}}\\ \Leftrightarrow t_{i,q,k}\left(\frac{h_{i,q,k}^{t}t_{i,q,k}}{I_{-i,q,k}}-\gamma_{\mathrm{th}}^{\min}\right)-\frac{h_{i,q,k}^{t}\gamma_{\mathrm{th}}^{\min}}{\eta_{i,q,k}\frac{\gamma_{i,q,k}}{t_{i,q,k}}-\mu_{i,q,k}+\psi_{i,q,k}}=0 \end{array}$$

After simplification, the optimal solution $t_{i,q,k}^{*}$ of the $i^{\mathrm{th}}$ radar dwell time is obtained as follows:

(26) $$t_{i,q,k}^{*}=\frac{1}{2}\left(\frac{I_{-i,q,k}}{h_{i,q,k}^{t}}\gamma_{\mathrm{th}}^{\min}+\sqrt{A^{*}}\right)$$

where:

(27) $$A^{*}={\left(\frac{I_{-i,q,k}}{h_{i,q,k}^{t}}\gamma_{\mathrm{th}}^{\min}\right)}^{2}+\frac{4\gamma_{\mathrm{th}}^{\min}I_{-i,q,k}^{2}}{h_{i,q,k}^{t}\eta_{i,q,k}^{*}+\left[\psi_{i,q,k}^{*}-\mu_{i,q,k}^{*}\right]I_{-i,q,k}}$$

With the help of Newton iterative method, the iterative formula of dwell time is obtained as follows:

(28) $$t_{i,q,k}^{\left(l+1\right)}={\left[\frac{1}{2}\left(\frac{t_{i,q,k}^{\left(l\right)}}{\gamma_{i,q,k}^{\left(l\right)}}\gamma_{\mathrm{th}}^{\min}+\sqrt{B^{\left(l\right)}}\right)\right]}_{T^{\min}}^{T^{\max}}$$

where:

(29) $$B^{\left(l\right)}={\left(\frac{t_{i,q,k}^{\left(l\right)}}{\gamma_{i,q,k}^{\left(l\right)}}\gamma_{\mathrm{th}}^{\min}\right)}^{2}+\frac{4\gamma_{\mathrm{th}}^{\min}h_{i,q,k}^{t}{\left(\frac{t_{i,q,k}^{\left(l\right)}}{\gamma_{i,q,k}^{\left(l\right)}}\right)}^{2}}{\eta_{i,q,k}^{\left(l\right)}+\left[\psi_{i,q,k}^{\left(l\right)}-\mu_{i,q,k}^{\left(l\right)}\right]\left(\frac{t_{i,q,k}^{\left(l\right)}}{\gamma_{i,q,k}^{\left(l\right)}}\right)}$$

where l is the index of iteration times. The subgradient algorithm is used to update the Lagrange multipliers ${\left\{{\left(\eta_{i,q,k}\right)}^{\left(l\right)}\right\}}_{i=1}^{N}$, ${\left\{{\left(\mu_{i,q,k}\right)}^{\left(l\right)}\right\}}_{i=1}^{N}$, and ${\left\{{\left(\psi_{i,q,k}\right)}^{\left(l\right)}\right\}}_{i=1}^{N}$ in the dwell time allocation algorithm proposed in algorithm 1 to ensure the fast convergence of the algorithm:

(30) $$\left\{\begin{array}{l} \eta_{i,q,k}^{\left(l+1\right)}={\left[\eta_{i,q,k}^{\left(l\right)}-s_{t}\left(\gamma_{i,q,k}^{\left(l\right)}-\gamma_{\mathrm{th}}^{\min}\right)\right]}_{0}^{+}\\ \mu_{i,q,k}^{\left(l+1\right)}={\left[\mu_{i,q,k}^{\left(l\right)}-s_{t}\left(T^{\mathrm{man}}-t_{i,q,k}^{\left(l\right)}\right)\right]}_{0}^{+}\\ \psi_{i,q,k}^{\left(l+1\right)}={\left[\psi_{i,q,k}^{\left(l\right)}-s_{t}\left(t_{i,q,k}^{\left(l\right)}-T^{\min}\right)\right]}_{0}^{+} \end{array}\right.$$

where $s_{t}$ is the iteration step size, $l\in \left\{1,\cdots ,L_{\max}\right\}$ and $L_{\max}$ is the maximum number of iterations of the algorithm. It can be seen from Equation (30) that the Lagrange multipliers ${\left\{{\left(\eta_{i,q,k}\right)}^{\left(l\right)}\right\}}_{i=1}^{N}$, ${\left\{{\left(\mu_{i,q,k}\right)}^{\left(l\right)}\right\}}_{i=1}^{N}$, and ${\left\{{\left(\psi_{i,q,k}\right)}^{\left(l\right)}\right\}}_{i=1}^{N}$ can be updated by local iteration. The iterative algorithm flow of dwell time allocation based on NBS is shown in algorithm 1.

According to algorithm 1, the dwell time of each radar can be calculated iteratively according to Equation (17) under the condition of satisfying detection performance constraints. After a limited number of bargaining games, when the dwell time of each radar no longer changes, the optimal dwell time strategy ${\left\{t_{i,q,k}^{*}\right\}}_{i=1}^{N}$ that meets the model optimization target q is obtained, that is, the payment set obtained by the game participants after cooperation. In order to meet the calculation requirements of Algorithm 1, each radar needs to obtain the variances ${\left\{h_{i,q,k}^{t}\right\}}_{i=1}^{N}$ and ${\left\{h_{i,j,k}^{d}\right\}}_{j=1,j\neq i}^{N}$ of each path gain in advance.
**Algorithm 1** Dwell time allocation algorithm based on the Nash bargaining solution (NBS)**Step 1**: Parameter initialization: At the $k^{\mathrm{th}}$ time instant, for q=1,⋯,Q, set the parameter initial values $\gamma_{\mathrm{th}}^{\min}$, $T^{\min}$ and $T^{\max}$, Lagrangian multipliers ${\left\{{\left(\eta_{i,q,k}\right)}^{\left(l\right)}\right\}}_{i=1}^{N}$
$,\;{\left\{{\left(\mu_{i,q,k}\right)}^{\left(l\right)}\right\}}_{i=1}^{N}$ and ${\left\{{\left(\psi_{i,q,k}\right)}^{\left(l\right)}\right\}}_{i=1}^{N}$ , the number of iteration index l=1, error tolerance ε>0;

**Step 2**: Circulation: At the $k^{\mathrm{th}}$ time instant, for q=1,⋯,Q, use Equation (28) to calculate $t_{i,q,k}^{\left(l\right)};$ Use Equation (30) to update Lagrange multipliers; Update l←l+1; **Step 3**: When $\left|t_{i,q,k}^{\left(l+1\right)}-t_{i,q,k}^{\left(l\right)}\right|<\varepsilon$ or $l=L_{\max}$, end the cycle; **Step 4:** Repeat Parameter update: For ∀i, update $t_{i,q,k}^{*}\leftarrow t_{i,q,k}^{\left(l\right)}.$

### 3.3. Radar Selection Optimization

Through the cooperative game, the optimal solution of the dwell time of each target under the given radar assignment indicators can be obtained. By solving Equation (22) Q times, the optimal dwell time solution of radar combinations satisfying the constraint condition $\sum_{i=1}^{N}v_{i,q,k}^{}=1$ for Q targets can be obtained. The fixed Hungarian algorithm can be used to get the optimal results of the dwell time and radar allocation indicators, which meet the constraint conditions $\sum_{q=1}^{Q}v_{i,q,k}^{}\leq 1$. Assuming that $t_{i,q,k,\min}^{}$ represents the minimum dwell time of the $i^{\mathrm{th}}$ radar irradiating the $q^{\mathrm{th}}$ target at the $k^{\mathrm{th}}$ time instant, the minimum dwell time matrix $t_{k,\min}^{}$ at the $k^{\mathrm{th}}$ time instant composed of $t_{i,q,k,\min}^{}$ is shown in Table 1.

The optimization model of radar allocation indicators can be described as follows:

(31) $$\begin{array}{ccc} \min\sum_{q=1}^{Q}\sum_{i=1}^{N}v_{i,q,k}^{}t_{i,q,k,\min} & s.t. & \left\{\begin{array}{c} \sum_{q=1}^{Q}v_{i,q,k}^{}\leq 1\\ \sum_{i=1}^{N}v_{i,q,k}^{}=1 \end{array}\right. \end{array}$$

Equation(31) is an unbalanced assignment problem, which can be solved by the fixed Hungarian algorithm [27]. The optimal result of radar assignment indicators can be obtained by using Algorithm 2.
**Algorithm 2** Radar allocation method**Step 1:** Solve the Equation (22) Q times to obtain the minimum dwell time matrix $t_{k,\min}^{}\in {\mathbb{Z}}^{N\times Q}$ satisfying $\sum_{i=1}^{N}v_{i,q,k}^{}=1.$
**Step 2:** Arrange the columns of matrix $t_{k,\min}^{}$ in ascending order, and assign the target corresponding to the smallest element in the first row to the corresponding radar combination. **Step 3:** Remove the column vector corresponding to the target assigned in step 2, and remove all row vectors containing the radar in the radar combination assigned in step 2. **Step 4:** Repeat steps 2 and 3 until all targets are assigned to get the optimal allocation of radar combination.

## 4. Performance Verification

### 4.1. Simulation Settings

In order to verify the effectiveness of the multiple target tracking dwell time optimization algorithm based on cooperative game, the number of radars in radar network is assumed to be N=6 and the number of targets is Q=2. In this paper, it is assumed that each radar is in working state to calculate dwell time and allocate the radar. The missed detection probability $p_{i,q,k}^{\mathrm{MD}}\left(\delta_{i,q,k},\gamma_{i,q,k}\right)=0.0027$, the false alarm probability $p_{i,q,k}^{\mathrm{FA}}\left(\delta_{i,q,k}\right)=10^{-6}$, the detection threshold $\delta_{i,q,k}=0.0267$, and the corresponding SINR threshold $\gamma_{\mathrm{th}}^{\min}=10\;\mathrm{dB}$. Radar parameter settings are shown in Table 2.

**Figure 1:**
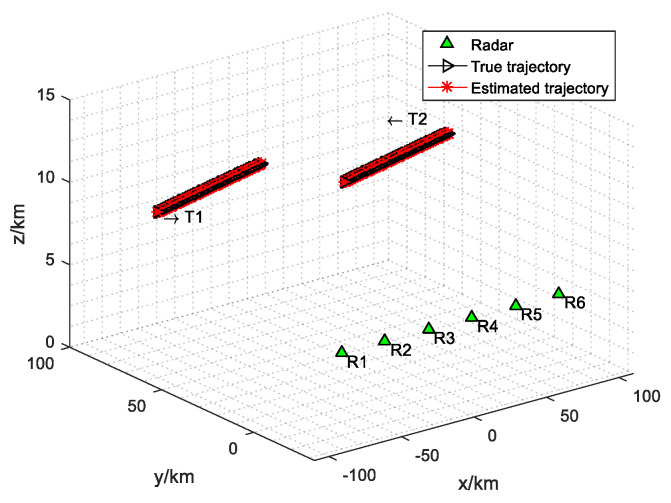
Schematic diagram of the target trajectory and radar network in the simulation scene.

The initial position of target 1 is (−100, 60, 6) km, flying at constant speed (300, 50, 0) m/s, and the initial position of target 2 is (100, 60, 6) km, flying at a constant speed of (−300, −50, 0) m/s. Suppose that the sampling interval of radar network is T=3 s, and the tracking process duration is 300 s. The maximum dwell time is $T_{}^{\max}=0.1\;s$ and the minimum value is equal to the radar pulse repetition period $T_{r}$. The convergence value of dwell time does not depend on the choice of Lagrange multipliers. Different Lagrange multipliers only affect the convergence speed of the algorithm. Set the maximum iteration number $L_{\max}=30$, Lagrange multipliers $\eta_{i,q,k}^{\left(0\right)}=10$, $\mu_{i,q,k}^{\left(0\right)}=10$, and $\psi_{i,q,k}^{\left(0\right)}=10$, error tolerance $\varepsilon =10^{-15}$, and iteration step size $s_{t}=0.001$. The array layout and target trajectory of radar network are shown in Figure 1. Where $T_{1}$ and $T_{2}$ are target 1 and target 2, $R_{1}\cdots R_{6}$ are radar 1 to radar 6. It can be calculated that $C_{1}=2.5407$.

Firstly, the dwell time allocation algorithm and radar selection problem based on the cooperative game are studied. Secondly, the relationship between the parameters of radar network to each target is explained when the algorithm proposed in this paper is used to track the target. Thirdly, the convergence of game iterations in the dwell time allocation algorithm based on the cooperative game is discussed and analyzed.

### 4.2. Simulation Results

In order to analyze the influence of different factors on the result of radar dwell time allocation, the dwell time allocation ratio of radar network is given. The dwell time allocation ratio of the $i^{\mathrm{th}}$ radar for the $q^{\mathrm{th}}$ target is defined as:

(32) $$\eta_{i}=\frac{t_{i,q,k}}{\sum_{i=1}^{N}t_{i,q,k}}$$

**Figure 2:**
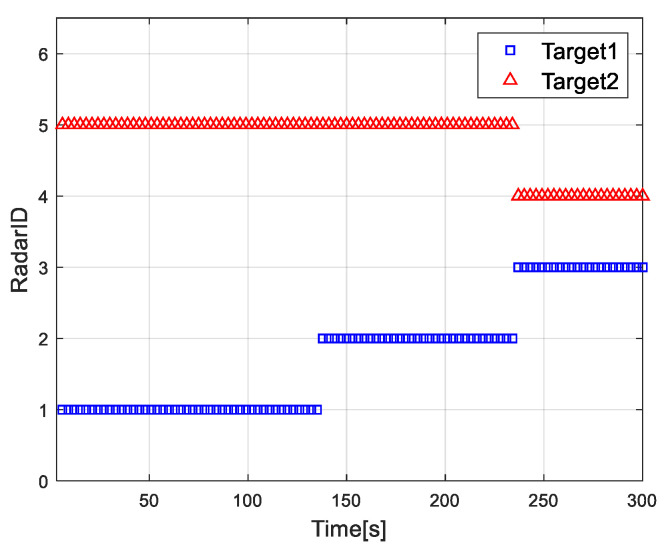
Assignment result between radar and target in radar network.

**Figure 3:**
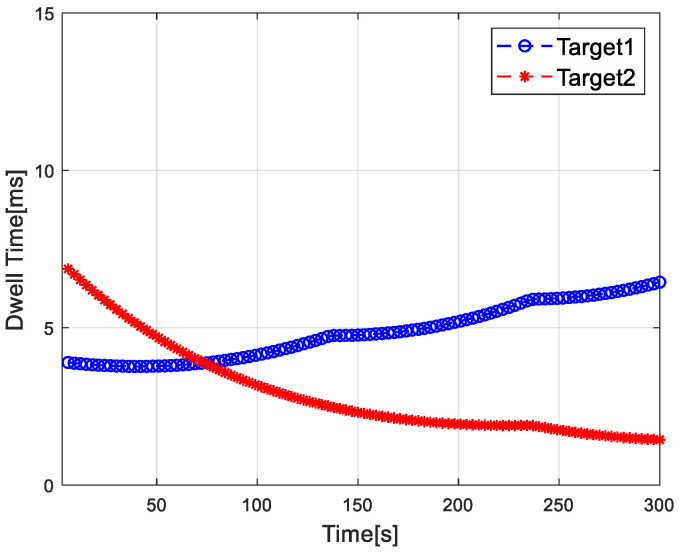
Dwell time of radar network on target.

**Figure 4:**
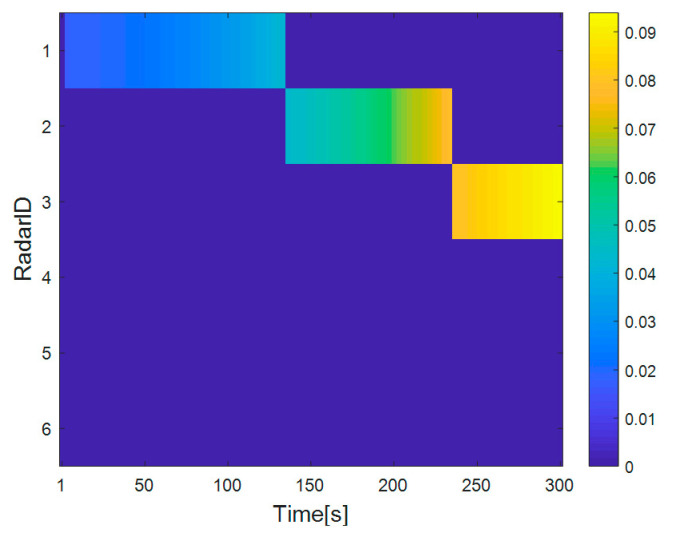
Target 1 dwell time allocation ratio.

**Figure 5:**
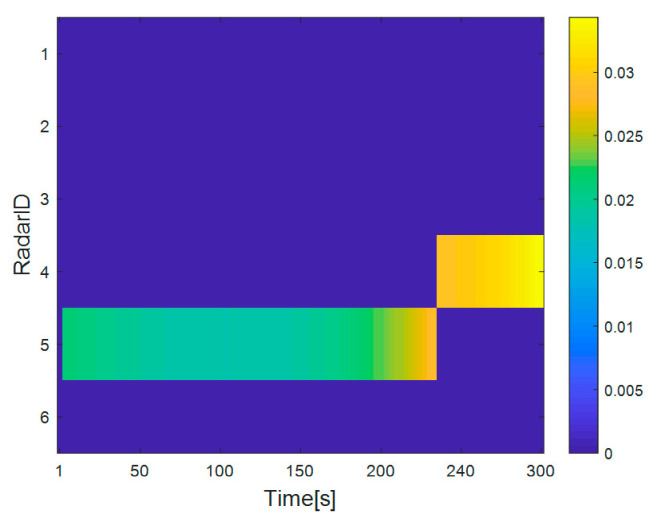
Target 2 dwell time allocation ratio.

Figure 2 shows the distribution results of each radar and target in the radar network. Figure 3 shows the dwell time of the radar network to each target at each time when the proposed algorithm tracks the target. Figure 4 and Figure 5 show the radar dwell time allocation ratio of target 1 and target 2, respectively. As can be seen from Figure 2 and Figure 4, taking target 1 as an example, in the first 138 s, the distance between target 1 and radar 1 is relatively close, which is illuminated by radar 1; from 138 s, target 1 is close to radar 2, and until 234 s it is used to irradiate target 1; from 235 s, target 1 is close to radar 3, and until 300 s, radar 3 irradiates it. In the tracking process, with the movement of the target, the radar close to the target will be given priority. It can be seen from Figure 3 that the dwell time of target 1 increases with the distance away, while the dwell time of target 2 decreases with the distance approaching.

In order to analyze the impact of dwell time optimization algorithm on LPI performance of radar network, the dwell time performances of the four dwell time control algorithms are compared in Table 3. The four algorithms are as follows: (1) the proposed algorithm; (2) the fixed dwell time radar assignment (FDTARA) algorithm; (3) Bayesian Cramerér-Rao lower bound-genetic algorithm (BCRLB-GA) [28,29]; (4) the adaptive non-cooperative dwell time control (ANCDTC) algorithm [30]. It can be seen from Table 3 that when the algorithm proposed in this paper is used for tracking, the total dwell time of radar network to all targets is the least, which is far lower than FDTARA tracking and ANCDTC algorithm tracking. It should be pointed out that BCRLB-GA ignores the interference constraints and optimizes the dwell time allocation in radar network without considering harmful interference. This algorithm is superior to ANCDTC algorithm in dwell time control. ANCDTC algorithm consumes much longer dwell time than the algorithm proposed in this paper, because each participant maximizes its utility function in a selfish and rational way. In order to better illustrate the optimization effect of this algorithm in radar network, the statistics of target 3 are added in Table 3. The initial position of target 3 is (100, 60, 6)km, flying at constant speed (300, 50, 0)m/s. Target 3 is far away from the radar network system, while target 1 and target 2 move in the radar network system. The dwell time of target 1 and target 2 is less than that of target 3 after optimized allocation of radar networking system.

**Figure 6:**
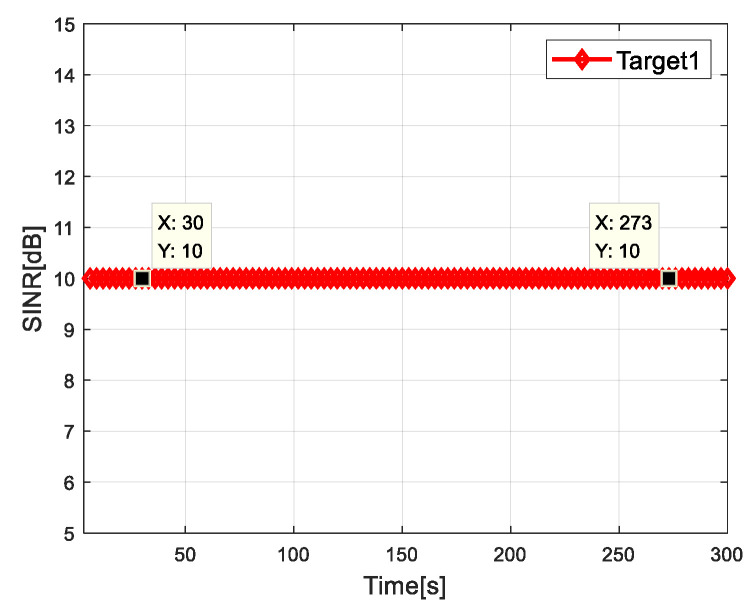
SINR for target 1.

**Figure 7:**
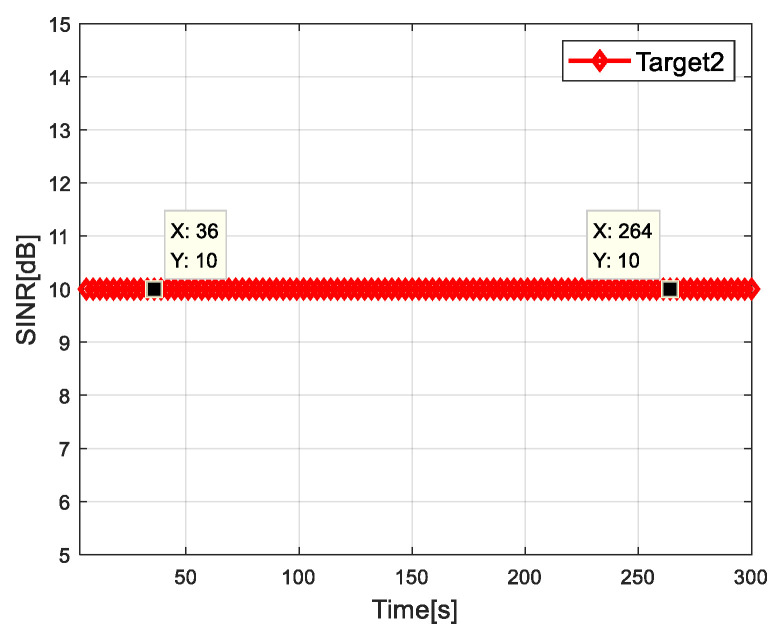
SINR for target 2.

Figure 6 and Figure 7 show the SINR of radar network to each target when tracking with the algorithm proposed in this paper. It can be seen that the SINR of all targets meets the requirements of threshold value. It is verified that the algorithm can control the dwell time of each radar and meet the target detection SINR performance.

**Figure 8:**
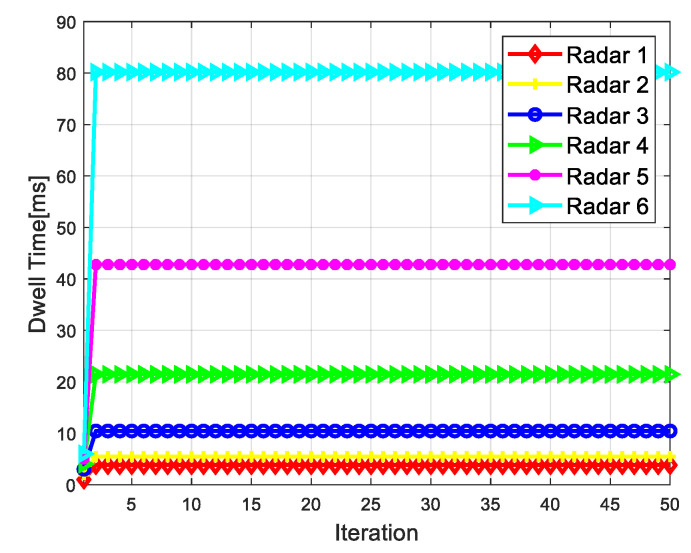
Radar dwell time convergence performance.

**Figure 9:**
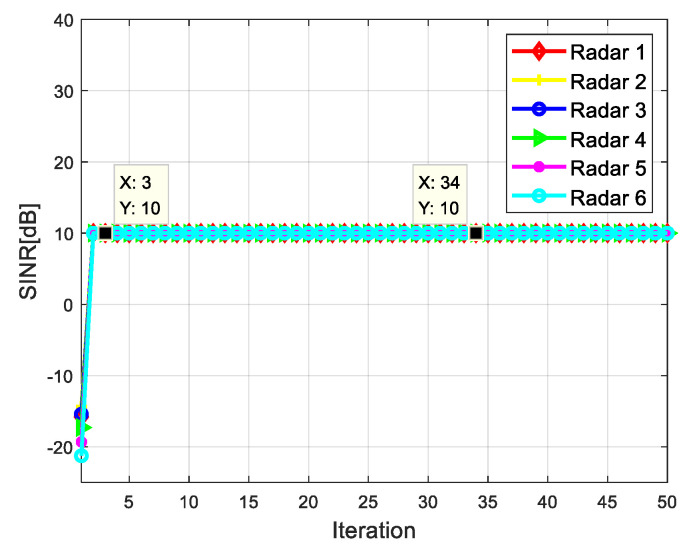
Radar SINR convergence performance.

**Figure 10:**
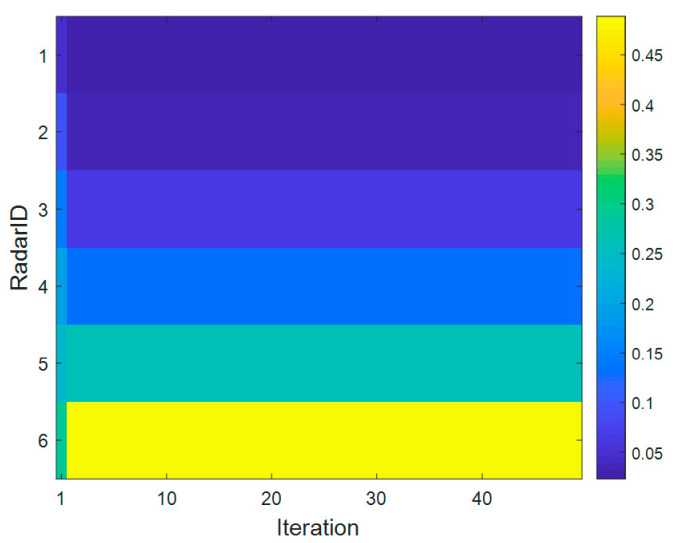
Target 1 radar dwell time allocation ratio.

Figure 8 shows the curve of radar dwell time varying with the number of game iterations in the dwell time allocation algorithm of radar network based on cooperative game. As can be seen from Figure 8, the proposed algorithm can reach Nash equilibrium point after iterative calculation, thus verifying the convergence of the algorithm. Figure 9 shows the SINR convergence performance of the dwell time allocation algorithm based on cooperative game. The results show that after the iterative calculation, the SINR of each radar converges to the SINR threshold $\gamma_{\mathrm{th}}^{\min}$. As shown in Figure 10, in the case of target 1, radar 1 and radar 2 allocate a smaller dwell time, while radar 5 and radar 6 allocate a larger dwell time, which indicates that the radar far from the target allocates a larger dwell time. The reason is that the farther the radar is from the target, the more dwell time is needed to meet the target detection SINR performance requirements. Therefore, the different location relationship of the target relative to each radar will produce different dwell time allocation results, which will affect the LPI performance of the radar network.

**Figure 11:**
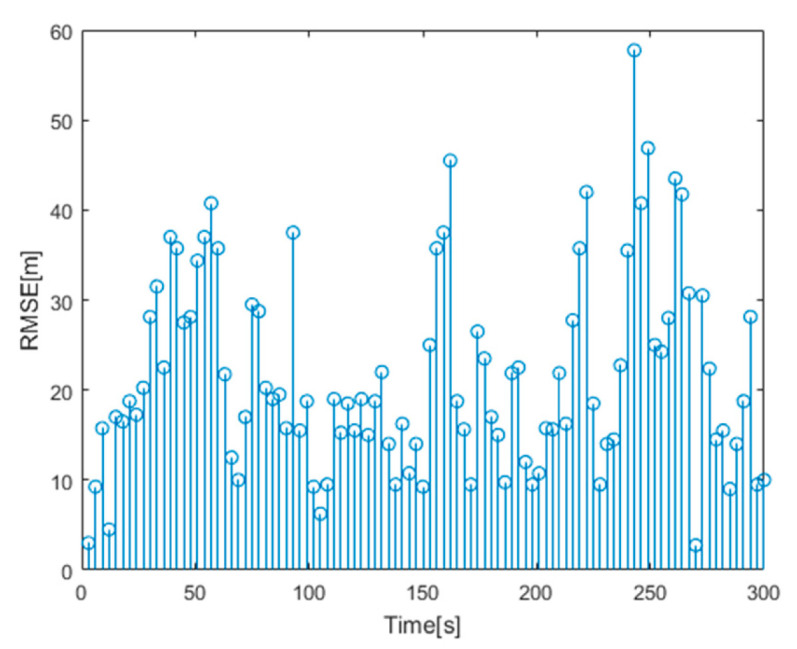
RMSE for target tracking.

The total tracking precision of all the targets at the $k^{\mathrm{th}}$ time instant is defined as Root Mean Square Error (RMSE):

(33) $$\mathrm{RMSE}\left(k\right)=\sum_{q=1}^{Q}\sqrt{\frac{1}{N_{\mathrm{MC}}}\sum_{l=1}^{N_{\mathrm{MC}}}\left\{{\left[x_{k}^{q}-{\hat{x}}_{l,k|k}^{q}\right]}^{2}+{\left[y_{k}^{q}-{\hat{y}}_{l,k|k}^{q}\right]}^{2}+{\left[z_{k}^{q}-{\hat{z}}_{l,k|k}^{q}\right]}^{2}\right\}}$$

where $N_{\mathrm{MC}}=100$ is the Monte Carlo experiment number and $\left({\hat{x}}_{l,k|k}^{q},{\hat{y}}_{l,k|k}^{q},{\hat{z}}_{l,k|k}^{q}\right)$ is the target position estimation obtained in the $n^{\mathrm{th}}$ Monte Carlo experiment. Figure 11 is the RMSE tracked by using the proposed algorithm.

The radar network arrangements for different numbers of radars are as follows: (1) N=2: Radar 3 and Radar 4; (2) N=3: Radar 3, Radar 4, and Radar 5; (3) N=4: Radar 2, Radar 3, Radar 4, and Radar 5; (4) N=5: Radar 1, Radar 2, Radar 3, Radar 4, and Radar 5; (5) N=6: Radar 1, Radar 2, Radar 3, Radar 4, Radar 5, and Radar 6.

**Figure 12:**
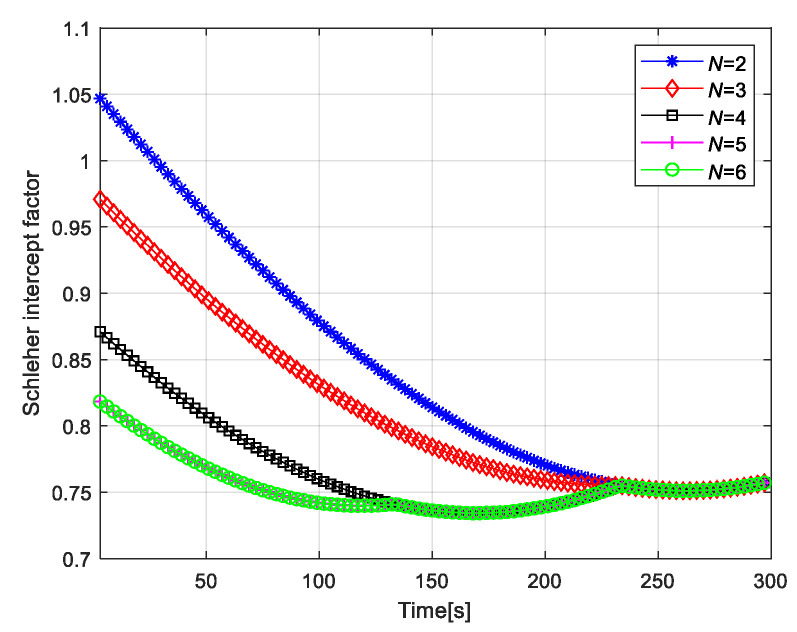
Total Schleher intercept factor.

Figure 12 shows the Schleher interception factor of radar network by using different number of radars for target tracking. When the N=6 radar network is used to track the target, a better Schleher intercept factor can be obtained. From the time index 1s to 134s, when N=6 and N=5 are adopted, radar 1 which is close to target 1 is used for tracking. Compared with N=4, 3, and 2, the Schleher interception factors of N=6 and N=5 radar networks are smaller. From 135 s to 234 s, the radar networks of N=6, 5, and 4 track target 1 with radar 2 closer to target 1, while radar 3, which is far away from target 1, is used to track target 1 for N=3 and 2 radar networks. Moreover, when the radar networks of N=3 and N=2 track target 2, radar 5, which is closer to target 2, is used. Therefore, the radar networks of N=3 and 2 have a poor Schleher interception factor during this period. The results show that better LPI optimization results can be obtained when there are close radars in the radar network, and vice versa. That is to say, the difference of LPI performance of radar network depends on the geometric layout of radar network, the number of radars, and the distance between target and radar network.

**Figure 13:**
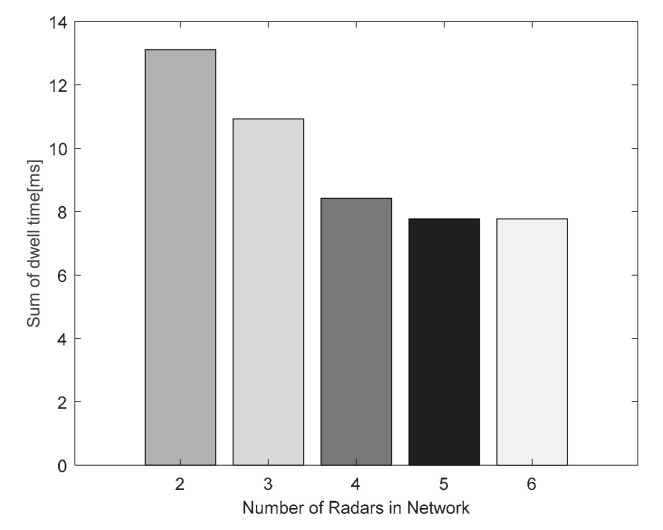
Comparison of total dwell time of radar network with different numbers of radars.

It can be seen from Figure 13 that the total dwell time of N=6 and N=5 radar networks is at a minimum, because the sixth radar is far away from target 2 and is not assigned to track target 2. When N=2 radar network is used to track the target, the total dwell time is the largest. The total dwell time of N=3 radar network is less than that of N=2 radar network, and is greater than that of N=4 radar network. Therefore, the number of radars and the geometry of radar network will affect the total dwell time. The algorithm and the radar network form proposed in this paper are used to balance the tracking target’s total dwell time with the radar network LPI performance.

## 5. Conclusions

In this paper, aiming at the problem of LPI in the radar network system, cooperative game theory and NBS were applied to optimize LPI performance of radar network target tracking, and a radar network dwell time allocation model based on NBS is proposed. Our primary objective was to minimize the total dwell time consumption of the radar network system, while guaranteeing each radar’s target detection requirement. The algorithm can achieve Pareto optimality of the system and fairness among radars by optimizing the dwell time of each radar under the condition of satisfying the detection performance constraints. The dwell time allocation problem of radar network based on NBS was transformed into a classical optimization problem. The optimal dwell time strategy of each radar was obtained by Lagrange relaxation algorithm, and the dwell time iteration formula of each radar was obtained by combining Newton iteration method. According to the results of dwell time allocation, the fixed Hungarian algorithm was used to select the target allocation schemes. Compared with the comparison algorithm, the proposed algorithm can greatly shorten the total dwell time of all targets irradiated by the radar network in the tracking process, effectively reduce the total Schleher intercept factor of radar network, and ensure the detection performance of all target tracking. The results also showed that the geometric arrangement of radar network, the distance between the target and radar network, and the number of radars can affect the total Schleher interception factor of the radar network.

## Figures and Tables

**Table 1:** Minimum dwell time matrix.

Minimum Dwell Time		Target No.			
		1	2	⋯	Q
**Radar No.**	1	$t_{1,1,k,\min}^{}$	$t_{1,2,k,\min}^{}$	⋯	$t_{1,Q,k,\min}^{}$
	2	$t_{2,1,k,\min}^{}$	$t_{2,2,k,\min}^{}$	⋯	$t_{2,Q,k,\min}^{}$
	⋮	⋮	⋮	⋯	⋮
	N	$t_{N,1,k,\min}^{}$	$t_{N,2,k,\min}^{}$	⋯	$t_{N,Q,k,\min}^{}$

**Table 2:** Radar Parameter.

Parameter	Value	Parameter	Value
Transmitted gain $G_{t}$	27 dB	Pulse repetition interval $T_{r}$	$1\times 10^{-5}\;s$
Received gain $G_{r}$	27 dB	Wavelength λ	0.01 m
Transmitted side lobe gain $G_{t}^{\prime }$	−30 dB	Correlation coefficient $C_{i,j,k}$	$10^{-4}$
Received side lobe gain $G_{r}^{\prime }$ Transmitted power $p_{i,q,k}$	−30 dB 1000 W	Background noise power $\sigma^{2}$ Pulses number n	$10^{-18}\;W$ 512

**Table 3:** Comparison of total dwell time of radar network.

	Target 1	Target 2	Target 3	All of the Targets
Proposed Algorithm	0.53 s	0.35 s	0.61 s	1.49 s
ANCDTC	2.27 s	1.5 s	2.61 s	6.38 s
BCRLB-GA	0.55 s	0.38 s	0.66 s	1.59 s
FDTARA	10 s	10 s	10 s	30 s
